# Perfluorooctanoic acid–associated complement factor B upregulation and atrial fibrillation–related molecular alterations: integrated network toxicology, Mendelian randomization, molecular dynamics, and HL-1 cell analyses

**DOI:** 10.3389/fcvm.2026.1759840

**Published:** 2026-09-03

**Authors:** Dan Li, Chuanfeng Bai, Jinping Zhao

**Affiliations:** 1 Department of Cardiovascular Surgery, Zhongnan Hospital of Wuhan University, Wuhan, Hubei, China; 2 Department of Cardiovascular Surgery, Hubei Provincial Engineering Research Center of Minimally Invasive Cardiovascular Surgery, Wuhan, Hubei, China; 3 Department of Cardiovascular Surgery, Wuhan Clinical Research Center for Minimally Invasive Treatment of Structural Heart Disease, Wuhan, Hubei, China; 4 Department of Pediatric Oncology, Shandong Cancer Hospital and Institute, Shandong First Medical University and Shandong Academy of Medical Sciences, Jinan, China

**Keywords:** atrial fibrillation, cardiotoxicity, complement factor B, environmental pollutant, perfluorooctanoic acid

## Abstract

**Background:**

Perfluorooctanoic acid (PFOA) is a persistent environmental pollutant associated with cardiovascular dysfunction, but the molecular links between PFOA exposure and atrial fibrillation (AF) remain unclear. We aimed to prioritize a candidate mediator connecting PFOA with AF and examine its associated molecular effects in atrial cardiomyocytes.

**Methods:**

PFOA-associated or computationally predicted genes from CTD, STITCH, and SwissTargetPrediction were integrated with AF-associated genes. Complement factor B (CFB) was evaluated using exploratory Mendelian randomization, public single-cell analysis, protein-interaction and enrichment analyses, molecular docking, a single 100-ns molecular dynamics trajectory, and HL-1 cell experiments. Cells were exposed to 25 μM PFOA for 48 h with or without CFB silencing, followed by qRT-PCR, Western blotting, immunofluorescence, and CCK-8 assays.

**Results:**

CFB was the only shared candidate between the integrated PFOA-related target set and the filtered AF-associated gene set. Genetically predicted plasma CFB levels were positively associated with AF risk (odds ratio = 1.109, 95% confidence interval: 1.021–1.205; FDR-adjusted *P* = 0.015). Public single-cell data showed preferential basal CFB expression in cardiac fibroblast and stromal populations, with lower expression in cardiomyocytes. The predicted PFOA–CFB pose remained within the selected pocket during the sampled trajectory. In HL-1 cells, PFOA increased CFB mRNA and protein abundance, decreased CACNA1C transcript levels without significantly changing CACNA1C protein abundance, and increased KCNJ2 and NPPA expression. CFB silencing attenuated the PFOA-associated increases in KCNJ2 and NPPA, whereas CACNA1C showed no corresponding rescue pattern. CFB immunoreactivity was detected within cTnT-positive, PFOA-exposed HL-1 cells.

**Conclusion:**

These findings prioritize CFB as a candidate molecule potentially associated with PFOA exposure and AF-related molecular alterations. They do not establish direct PFOA–CFB binding, functional complement activation, causality, electrical remodeling, or a functional AF phenotype.

## 1 Introduction

Perfluorooctanoic acid (PFOA), a member of the per- and polyfluoroalkyl substances (PFAS) family, is a synthetic compound widely used in industrial applications and consumer products such as non-stick coatings, firefighting foams, and water-repellent textiles (1, 2). Its carbon–fluorine backbone makes it highly resistant to environmental degradation and biological metabolism, contributing to its persistence in water, soil, and living organisms (3, 4). Human biomonitoring studies have shown that PFOA accumulates in blood and tissues, with a half-life exceeding three years in humans (5, 6). Accumulating evidence links PFOA exposure to multiple health outcomes, including developmental toxicity, endocrine disruption, metabolic dysfunction, and cancer (7–9). Of growing concern is the cardiovascular toxicity of PFOA. Population-based studies have reported associations between elevated serum PFOA levels and increased risk of hypertension, stroke, and cardiovascular mortality. For example, in the U.S. NHANES cohort, individuals with higher PFOA levels had significantly higher odds of cardiovascular disease (10, 11). Similar findings have been observed in PFAS-contaminated regions of Europe, reinforcing concerns over its long-term cardiovascular impact (12, 13).

Atrial fibrillation (AF) is the most common sustained cardiac arrhythmia, affecting millions of people worldwide (14, 15). It is characterized by disorganized electrical activity in the atria, leading to irregular and often rapid heart rhythm (16, 17). The development of AF involves both electrical and structural remodeling of atrial tissue, triggered or exacerbated by aging, hypertension, heart failure, and systemic inflammation (18, 19). Increasing evidence indicates that inflammation and immune activation play a pivotal role in the initiation and maintenance of AF. Inflammatory markers such as C-reactive protein (CRP), interleukin-6, and tumor necrosis factor-alpha are elevated in AF patients and are associated with recurrence and adverse outcomes (20, 21). Furthermore, immune components such as macrophage infiltration and NLRP3 inflammasome activation have been implicated in atrial remodeling and fibrosis (22, 23). Despite these advances, the role of complement activation—an essential arm of the innate immune response—remains understudied in the context of AF pathophysiology.

Complement factor B (CFB) is a central component of the alternative pathway of complement activation (24, 25). Upon binding with complement fragment C3b, CFB is cleaved by factor D to generate the C3 convertase (C3bBb), initiating a potent amplification loop that produces downstream effectors like C3a, C5a, and the membrane attack complex (26, 27). Dysregulation of this pathway is associated with inflammatory and cardiovascular diseases, yet its relevance in AF, especially in the context of environmental toxicant exposure, is unclear.

Network toxicology has emerged as a powerful approach to elucidate the complex interactions between environmental chemicals and disease phenotypes by integrating toxicogenomics, systems biology, and bioinformatics (28, 29). Unlike traditional toxicology, which often relies on high-dose animal experiments, network toxicology enables the identification of mechanistic pathways and molecular targets through data-driven inference across multi-omics platforms. Public resources such as the Comparative Toxicogenomics Database (CTD), STITCH, and SwissTargetPrediction provide different types of curated associations or computational predictions related to chemical–gene and chemical–protein relationships, enabling exploratory candidate prioritization and network construction. Integration with interaction databases like STRING and BioGRID facilitates enrichment analysis and identification of hub genes or signaling modules affected by specific toxicants. Such systems-level analyses are especially valuable for persistent pollutants like PFOA, which act through multiple low-affinity molecular interactions over time.

In this study, we integrated network toxicology, exploratory Mendelian randomization, public single-cell analysis, molecular docking and dynamics, and HL-1 cell experiments to prioritize and evaluate CFB as a candidate molecular link between PFOA exposure and AF. Specifically, we examined the association of genetically predicted plasma CFB levels with AF risk, characterized the cardiac expression and interaction landscape of CFB, explored a predicted PFOA–CFB binding pose, and assessed PFOA-associated changes in CFB and selected AF-related molecular markers in HL-1 atrial cardiomyocytes.

## 2 Materials and methods

### 2.1 Network toxicology analysis

#### 2.1.1 Collection of PFOA-associated candidate targets and AF-associated genes

The three-dimensional molecular structure of PFOA was retrieved from PubChem (https://pubchem.ncbi.nlm.nih.gov/) (30–32), and its predicted toxicological and physicochemical properties were obtained from ProTox. PFOA-associated or computationally predicted candidate targets were collected from the Comparative Toxicogenomics Database (CTD, https://ctdbase.org/) (33–35), STITCH (http://stitch.embl.de), and SwissTargetPrediction (https://www.swisstargetprediction.ch/) (36). Because these resources contain different types of curated associations and computational predictions, the collected genes were treated as candidate targets rather than uniformly interpreted as experimentally confirmed direct PFOA-binding targets. Gene symbols were standardized, duplicate entries were removed, and the integrated candidate set was used for subsequent exploratory network analyses.

Atrial fibrillation (AF)-associated genes were retrieved from GeneCards (https://www.genecards.org/) using the search term “atrial fibrillation” in December 2025, with the search restricted to Homo sapiens. The retrieved genes were ranked according to their GeneCards relevance scores. Because GeneCards does not provide a universally established disease-specific cutoff, genes with relevance scores greater than the median score of the complete retrieved gene set were retained for subsequent analysis.

**Figure 1:**
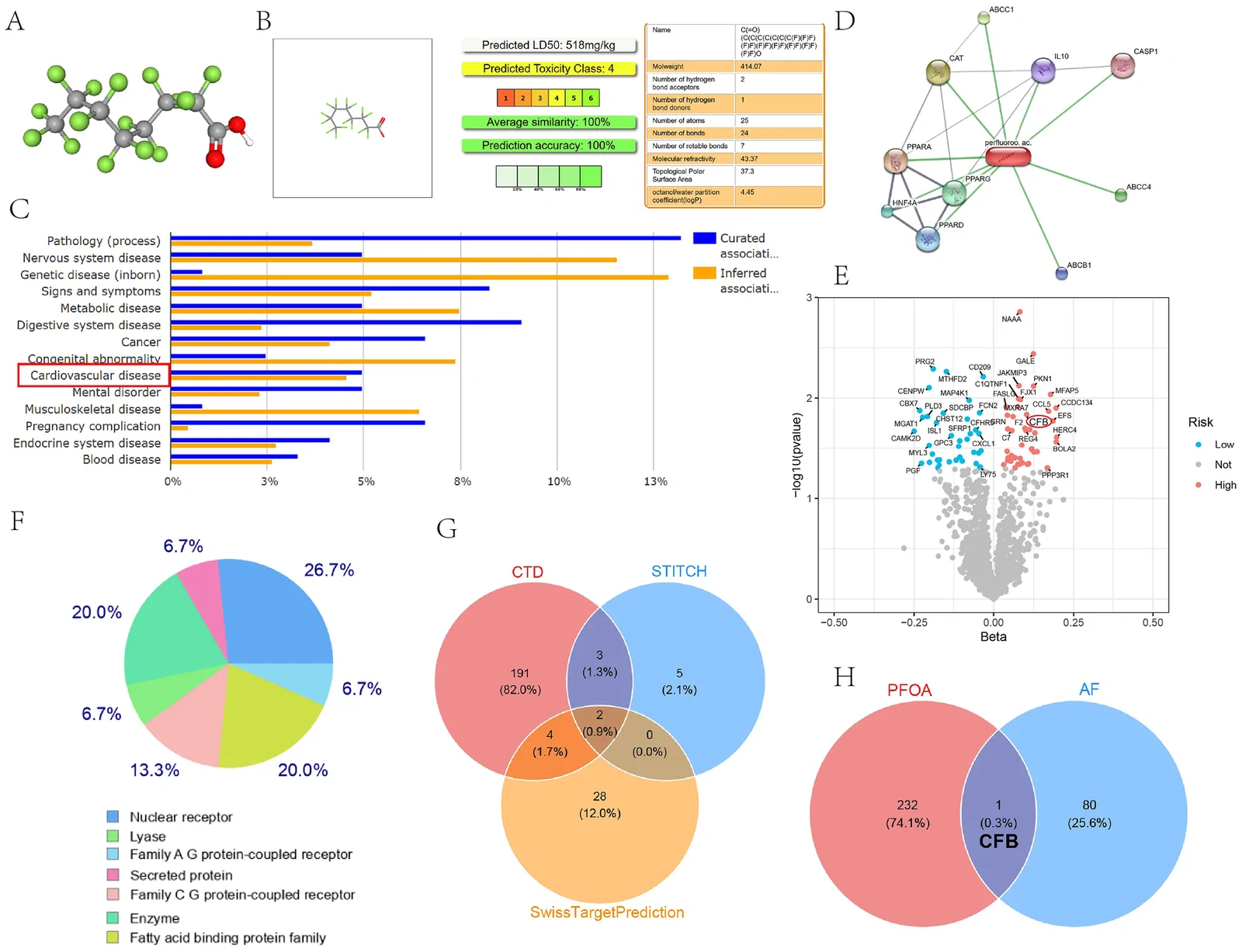
Network toxicology and exploratory Mendelian randomization analysis of PFOA. **(A)** Three-dimensional molecular structure of PFOA. **(B)** Predicted toxicological profile of PFOA obtained using the ProTox platform. **(C)** Disease categories associated with PFOA retrieved from the CTD database. **(D)** PFOA-associated candidate genes obtained from the STITCH database. **(E)** Volcano plot illustrating the exploratory Mendelian randomization results and highlighting the prioritized candidate protein CFB. **(F)** Computationally predicted candidate targets obtained from SwissTargetPrediction. **(G)** Source distribution of PFOA-associated or computationally predicted candidate targets obtained from CTD, STITCH, and SwissTargetPrediction after gene-symbol standardization and duplicate removal. **(H)** Intersection of the integrated PFOA-associated or computationally predicted candidate set and 81 AF-associated genes retained from GeneCards after median relevance-score filtering, HGNC-based gene-symbol standardization, and duplicate removal. CFB was identified as the only shared candidate for subsequent exploratory evaluation.

Gene aliases and previous gene symbols were converted to current HGNC-approved gene symbols. Non-human entries, records without valid official gene symbols, and duplicated genes were excluded. When multiple records corresponded to the same gene, the record with the highest relevance score was retained. After relevance-score filtering, gene-symbol standardization, and duplicate removal, 81 unique AF-associated genes were retained. These genes were intersected with the integrated PFOA-associated or computationally predicted candidate set, identifying CFB as the only shared candidate (Figure 1H). The intersection analysis was used solely for exploratory candidate prioritization and was not interpreted as evidence of direct PFOA–CFB binding, regulatory direction, or causality.

#### 2.1.2 Druggable target selection and Mendelian randomization analysis

PFOA-associated or computationally predicted candidate targets were first collected from CTD, STITCH, and SwissTargetPrediction as described above. To prioritize druggable candidates, these genes were intersected with druggable targets from DGIdb and the curated druggable genome list reported by Finan et al. A gene was retained as a candidate if it met both criteria: it was recorded as PFOA-associated or computationally predicted in at least one source resource and was classified as druggable in DGIdb or the druggable genome list reported by Finan et al. The full list of selected PFOA-related druggable targets and their supporting sources is provided in Supplementary Table S2.

Two-sample Mendelian randomization was used to explore associations between genetically predicted plasma levels of the prioritized candidate proteins and AF risk. Cis-pQTL data were obtained from the deCODE plasma proteome GWAS, and AF summary statistics were obtained from FinnGen release 12. The FinnGen endpoint was I9_AF, including atrial fibrillation and flutter, with 63,532 cases and 252,810 controls. Independent cis-pQTLs were selected after linkage-disequilibrium clumping, and allele harmonization was performed before analysis. The inverse-variance weighted method was used as the primary estimator, and heterogeneity was assessed using Cochran's *Q* test. Because comprehensive instrument-strength, horizontal pleiotropy, and leave-one-out sensitivity assessments were not performed, the MR results were interpreted as exploratory genetic associations rather than evidence of causality.

### 2.2 Gene expression analysis of CFB

To comprehensively investigate the expression characteristics of CFB in normal human tissues, we performed an integrated transcriptomic analysis using data from the Human Protein Atlas (HPA, https://www.proteinatlas.org/) (37, 38). The “Single Cell” and “Subcellular” sections of the HPA database were examined to characterize the cardiac cell-type-specific expression profile and immunofluorescence-based subcellular localization pattern of CFB. Heart single-cell RNA-seq data from the HPA single-cell module were reviewed using UMAP visualization, cell-type annotation, expression summaries, and marker-gene heatmaps to identify cardiac cell populations with detectable CFB expression. Subcellular localization of CFB was assessed using HPA immunofluorescence images generated with HPA-listed CFB antibodies, together with nuclear, microtubule, and endoplasmic reticulum markers. These images were obtained from the HPA database and were not experimentally generated in this study. In addition, the UCSC Cancer Genomics Browser (https://genome-cancer.ucsc.edu/) (39–41) was used to visualize the chromosomal location, transcript isoforms, exon–intron organization, OMIM annotation, common dbSNP variants, and RepeatMasker tracks of the CFB gene.

### 2.3 Molecular docking and molecular dynamics

The three-dimensional structure of human complement factor B (CFB) was obtained from the RCSB Protein Data Bank (PDB ID: 2OK5). The co-crystallized ligand in 2OK5 was retained as an internal positive control and referred to as “CFB-Original”. The structure of perfluorooctanoic acid (PFOA) was downloaded from PubChem and prepared as the deprotonated carboxylate form at physiological pH. Protein preparation included removal of crystallographic waters and non-specific heteroatoms, addition of polar hydrogens, and optimization of protonation states.

Molecular docking was performed using AutoDock Vina. PFOA was docked into the binding pocket surrounding the co-crystallized ligand site of CFB, with the grid box centered on the native ligand centroid. The top-ranked docking pose without steric clashes was selected as the initial conformation for the CFB–PFOA molecular dynamics (MD) simulation, while the crystallographic ligand pose was used for the CFB–Original control system.

MD simulations were conducted using GROMACS 2022.4. The AMBER14SB force field was used for CFB, and GAFF2 parameters with AM1-BCC charges were generated for PFOA and the co-crystallized ligand. Each system was solvated in a TIP4P water box, neutralized with Na⁺/Cl⁻ ions, and adjusted to 0.15 M ionic strength. After energy minimization, the systems were equilibrated under NVT and NPT ensembles at 310 K and 1 bar, followed by 100-ns production simulations with a 2-fs time step.

Trajectory analyses included backbone RMSD, residue-level RMSF, radius of gyration (Rg), hydrogen-bond formation, free-energy landscape analysis, representative structural snapshots, and short-range non-bonded interaction energies (Coul-SR and LJ-SR). The computational analysis was interpreted descriptively because only one 100-ns trajectory per system was analyzed and formal convergence validation was not performed.

### 2.4 Interactor collection and functional enrichment of CFB

Protein partners of Complement Factor B (CFB) were gathered from two complementary resources.

1. BioGRID (Homo sapiens) was queried for physical protein–protein interactions involving CFB. Genetic-only interactions, non-human entries, and redundant records were excluded.
2. STRING (Homo sapiens) was queried for CFB functional partners; only interactions with combined confidence score ≥0.70 (high confidence) were retained.

Interactor lists from the two databases were unioned to create a non-redundant set; UniProt accessions were mapped to Entrez Gene IDs and official HGNC symbols using AnnotationDbi and org.Hs.eg.db. Entries without a valid human Entrez ID were removed.

Over-representation analyses were performed in R (≥4.3) using clusterProfiler (v4.x).

KEGG pathways: enrichKEGG(organism=“hsa”, keyType=“kegg”) with parameters pvalueCutoff=0.05, pAdjustMethod=“BH”, qvalueCutoff=0.05, minGSSize=5, maxGSSize=500. The gene universe was all human Entrez IDs annotated in KEGG (default background in clusterProfiler).

Gene Ontology (GO): enrichGO(OrgDb=org.Hs.eg.db, keyType=“ENTREZID”, ont=“BP/CC/MF”, pvalueCutoff=0.05, pAdjustMethod=“BH”, qvalueCutoff=0.05, minGSSize=5, maxGSSize=500). Redundant GO terms were reduced with simplify() (similarity cutoff 0.7, method=“Wang”). Results were converted to readable symbols with setReadable().

Terms with Benjamini–Hochberg adjusted *p* value (p.adjust) < 0.05 were considered significant.

KEGG results were displayed as bar plots of gene counts with p.adjust encoded by a color gradient (panel C). GO results for BP/CC/MF were summarized as bar plots grouped by ontology with counts on the *y*-axis (panel D). Plots were generated with ggplot2/enrichplot; all counts represent the number of unique interactors annotated to each term. The BioGRID (panel A) and STRING (panel B) networks were visualized from the respective exports, with node size reflecting connectivity and edge thickness reflecting confidence (STRING).

### 2.5 Cell experiments

#### 2.5.1 Cell culture

The mouse atrial cardiomyocyte line HL-1 (Sigma-Aldrich, USA) was used as an *in vitro* atrial model. Culture vessels were pre-coated with 0.02% gelatin and fibronectin (5 µg/mL). Cells were maintained in Claycomb medium (Sigma) supplemented with 10% FBS, 2 mM L-glutamine, 0.1 mM norepinephrine (prepared fresh), and 1% penicillin–streptomycin, at 37 °C in a humidified 5% CO_2_ incubator. Medium was changed every 48 h and cells were passaged at 70%–80% confluence.

#### 2.5.2 Reagents and PFOA treatment

Perfluorooctanoic acid (PFOA) (MedChemExpress, USA) was dissolved as a 100 mM stock in DMSO and stored at −20 °C. Working solutions were prepared in culture medium immediately before use; the final DMSO concentration was ≤0.1% (v/v) in all groups. Based on the concentration- and time-response CCK-8 screening, 25 μM PFOA for 48 h was selected as an *in vitro* mechanistic condition because it did not significantly reduce CCK-8 viability under the tested conditions. This concentration was not intended to reproduce typical human serum exposure.

#### 2.5.3 siRNA transfection

For CFB knockdown, HL-1 cells were transfected with mouse Cfb-specific siRNA or negative-control siRNA (GenePharma, Shanghai, China) using Lipofectamine 3000 (Invitrogen, USA) in Opti-MEM (Gibco, USA) following the manufacturer's protocol. Cells were seeded in 6-well plates at 30%–40% confluence, and transfected with 50 nM siRNA. After 6 h, the transfection mixture was replaced with complete Claycomb medium and cells were incubated for 24–48 h before PFOA exposure. Knockdown efficiency was verified by qRT-PCR (and, where indicated, by Western blot).

#### 2.5.4 Cell viability assay (CCK-8)

HL-1 cells were seeded into 96-well plates at 5,000 cells/well and allowed to adhere overnight. Cells were treated with the indicated PFOA concentrations and durations (Section 2.5.2). At each time point, 10 µL CCK-8 reagent (Beyotime, China) was added per well and incubated at 37 °C for 1 h. Absorbance at 450 nm was read on a microplate reader (Thermo Fisher Scientific, USA). Background-subtracted OD values were normalized to the vehicle control. Each condition contained six technical replicates and three independent biological experiments.

#### 2.5.5 Quantitative real-time PCR (qRT-PCR)

Total RNA was extracted using TRIzol (Takara, Japan). cDNA was synthesized with Evo M-MLV RT Mix (Accurate Biology, China). qRT-PCR was performed on a LightCycler 480 II (Roche, Switzerland) using SYBR Green Premix Pro Taq HS (Accurate Biology). Primers targeted Cfb, CACNA1C, Kcnj2, and Nppa (mouse genes; synthesized by General Biosystems, Anhui, China). Gapdh served as the internal control. Relative expression was calculated by the 2^−ΔΔCt^ method. For experiments assessing the role of CFB, cells were assigned to Control, PFOA (25 µM), siCFB, and PFOA + siCFB groups.

#### 2.5.6 Western blot analysis

Western blotting was performed to assess CFB and selected AF-associated molecular markers at the protein level in HL-1 atrial cardiomyocytes. Cells were assigned to four groups: Control, PFOA, siCFB, and PFOA + siCFB, consistent with the qRT-PCR experiments. Protein expression of CFB, CACNA1C, KCNJ2, and NPPA was evaluated, with GAPDH used as the loading control.

Total protein was extracted from cells using RIPA lysis buffer supplemented with protease and phosphatase inhibitors. Protein concentrations were determined using a BCA protein assay kit. Equal amounts of protein were separated by SDS-PAGE and transferred onto PVDF membranes. After blocking with 5% non-fat milk or BSA at room temperature, membranes were incubated overnight at 4 °C with primary antibodies against CFB, CACNA1C, KCNJ2, NPPA, and GAPDH. All primary antibodies were purchased from Proteintech/Sanying Biotechnology. After washing, membranes were incubated with HRP-conjugated secondary antibodies, and protein bands were detected using an enhanced chemiluminescence substrate. GAPDH was used as the internal loading control. Band intensities were quantified using ImageJ software and normalized to GAPDH. Data are presented as mean ± SEM from at least three independent experiments. Statistical comparisons among multiple groups were performed using one-way ANOVA followed by *post-hoc* multiple-comparison testing, consistent with the statistical approach used in the cell experiments.

#### 2.5.7 Immunofluorescence staining

Immunofluorescence staining was performed to verify detectable CFB immunoreactivity within HL-1 atrial cardiomyocytes. HL-1 cells were seeded onto sterile coverslips in 24-well plates and treated with 25 μM PFOA for 48 h. Cells were washed, fixed, permeabilized, and blocked before incubation with primary antibodies against CFB and cardiac troponin T (cTnT). After incubation with species-appropriate fluorescent secondary antibodies, nuclei were counterstained with DAPI. Images were acquired using fluorescence microscopy under identical acquisition settings for the displayed channels. CFB was displayed in green, cTnT in red, DAPI in blue, and the merged CFB/cTnT signal in yellow where the channels overlapped.

#### 2.5.8 Statistics

Data are presented as mean ± SEM from ≥3 independent experiments. Multiple-group comparisons used one-way ANOVA followed by Tukey's *post-hoc* test. Significance thresholds: *p* < 0.05, *p* < 0.01, *p* < 0.001.

## 3 Results

### 3.1 Network toxicology analysis and Mendelian randomization of PFOA

To explore the potential molecular mechanisms linking perfluorooctanoic acid (PFOA) exposure to atrial fibrillation (AF), a network toxicology analysis was conducted. The three-dimensional molecular structure of PFOA is shown in Figure 1A. Protox-based prediction indicated moderate toxicity, with a predicted LD_50_ of 518 mg/kg and a toxicity class of 4 (Figure 1B). Disease enrichment analysis using the CTD database revealed that PFOA was mainly associated with metabolic, cardiovascular, and nervous system diseases (Figure 1C). PFOA-associated candidate genes obtained from the STITCH database were included in the exploratory network analysis (Figure 1D).

MR analysis was performed to evaluate the associations between PFOA-related druggable targets and AF risk. *P* values were adjusted using the Benjamini–Hochberg false-discovery-rate method, and proteins meeting the FDR-adjusted significance threshold were visualized in the volcano plot (Figure 1E). Among the prioritized candidates, CFB showed a significant positive association with AF risk using the IVW method (OR = 1.109, 95% CI: 1.021–1.205, FDR-adjusted *P* = 0.015; nsnp = 8). Heterogeneity analysis showed no significant heterogeneity for CFB (IVW Q = 2.175, *P* = 0.950; MR-Egger Q = 2.154, *P* = 0.905). Detailed MR statistics, including effect sizes, confidence intervals, FDR-adjusted *P* values, and heterogeneity results, are provided in Supplementary Table S1. SwissTargetPrediction classified the computationally predicted candidate targets into several protein categories, including enzymes, receptors, and transporters (Figure 1F). The targets collected from CTD, STITCH, and SwissTargetPrediction were standardized and merged to generate an integrated PFOA-related target set. Figure 1G shows the source distribution of these targets rather than a strict three-database overlap analysis, because the databases differ in evidence sources and prediction methods. To prioritize druggable candidates, the integrated PFOA-related targets were further intersected with druggable genes from DGIdb and the Finan druggable genome list. The selected druggable PFOA-related targets and their supporting sources are listed in Supplementary Table S2.

GeneCards searching in December 2025 identified AF-associated genes that were subsequently filtered using a predefined median relevance-score threshold. After gene-symbol standardization and removal of invalid and duplicated entries, 81 unique AF-associated genes were retained. Intersection of these genes with the integrated PFOA-associated or computationally predicted candidate set identified CFB as the only shared candidate for further exploratory evaluation (Figure 1H).

### 3.2 Cardiac single-cell distribution, subcellular localization, and genomic features of CFB

**Figure 2:**
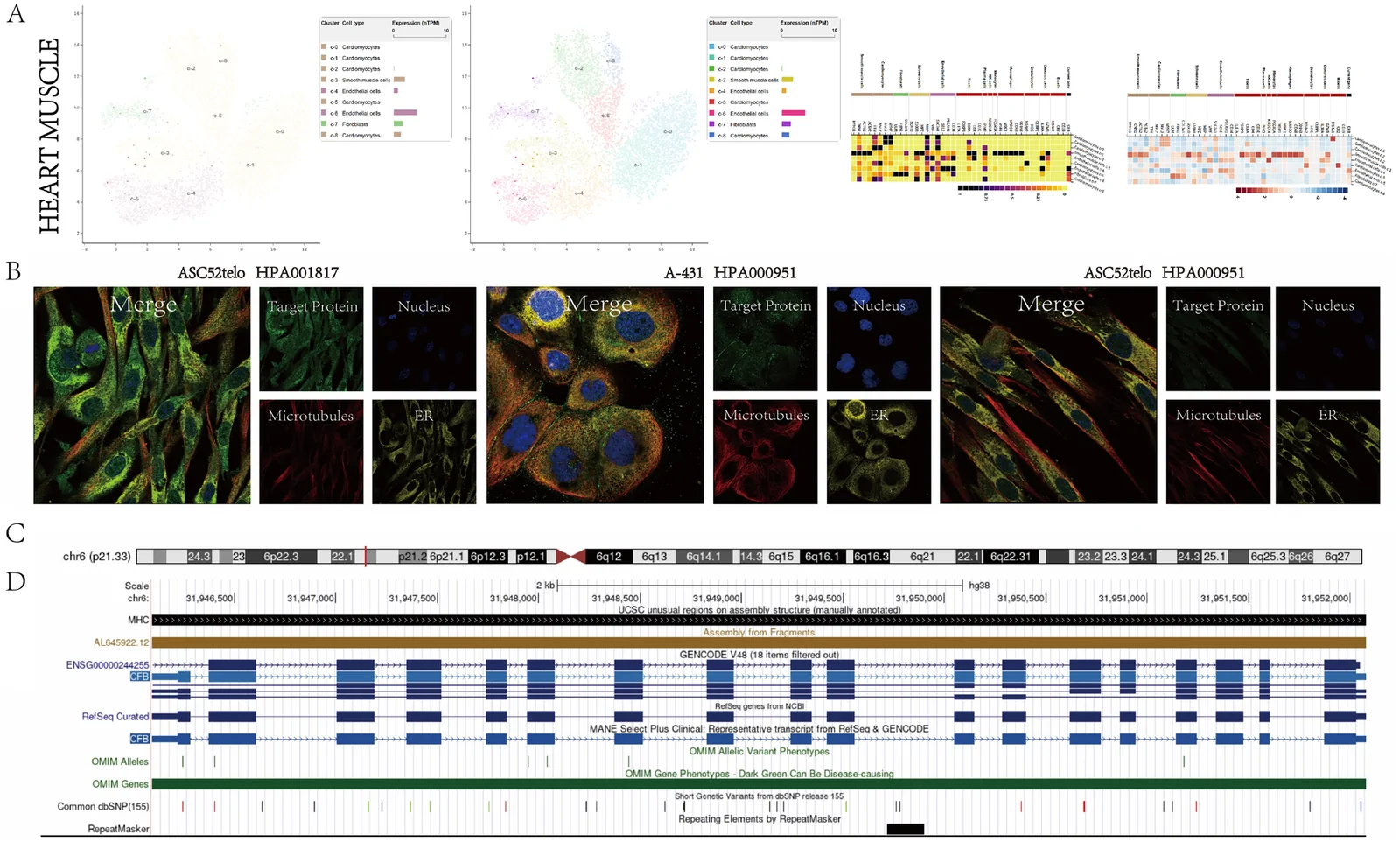
Cardiac single-cell distribution, subcellular localization, and genomic features of CFB. **(A)** HPA single-cell RNA-seq data showing preferential basal CFB expression in cardiac fibroblast and stromal populations, with relatively lower expression in cardiomyocyte and endothelial populations. **(B)** Representative HPA immunofluorescence images showing CFB-related signals in ASC52telo and A-431 cells. These publicly available images were used only for descriptive subcellular localization analysis. **(C)** Gene structure and chromosomal position of CFB on 6p21.33. **(D)** UCSC Genome Browser visualization showing annotated CFB transcript isoforms, exon–intron organization, OMIM annotation, common dbSNP variants, and RepeatMasker tracks.

To streamline the public database-based characterization of CFB, cardiac single-cell expression, subcellular localization, and genomic annotation were integrated into the revised Figure 2. HPA heart single-cell RNA-seq analysis showed that CFB expression was not broadly distributed across all cardiac cell types. Instead, detectable CFB signals were mainly observed in fibroblast and stromal-like cell populations, whereas cardiomyocytes showed relatively low expression (Figure 2A). These findings suggest that, although CFB is not highly expressed in bulk cardiac tissue, its cell-type-specific expression in non-cardiomyocyte populations may be relevant to local immune regulation, extracellular matrix remodeling, and complement-associated inflammatory responses in the cardiac microenvironment.

HPA immunofluorescence analysis further showed the subcellular distribution pattern of CFB. In ASC52telo and A-431 cells, CFB-related signals were observed at cell junctions and in vesicular/endoplasmic reticulum-associated compartments (Figure 2B). This localization pattern is consistent with the known role of CFB as a secreted complement component and supports its potential involvement in extracellular immune-related processes.

Genomic annotation showed that CFB is located on chromosome 6p21.33, within the major histocompatibility complex region (Figure 2C). UCSC Genome Browser visualization demonstrated multiple annotated transcript isoforms and exon-intron structures of CFB, together with OMIM, common dbSNP, and RepeatMasker tracks (Figure 2D). Collectively, these integrated analyses provide a concise overview of the cardiac cell-type distribution, subcellular localization, and genomic organization of CFB, supporting its biological relevance in complement-mediated inflammatory remodeling.

### 3.3 Molecular dynamics behavior of the predicted CFB–PFOA complex

**Figure 3:**
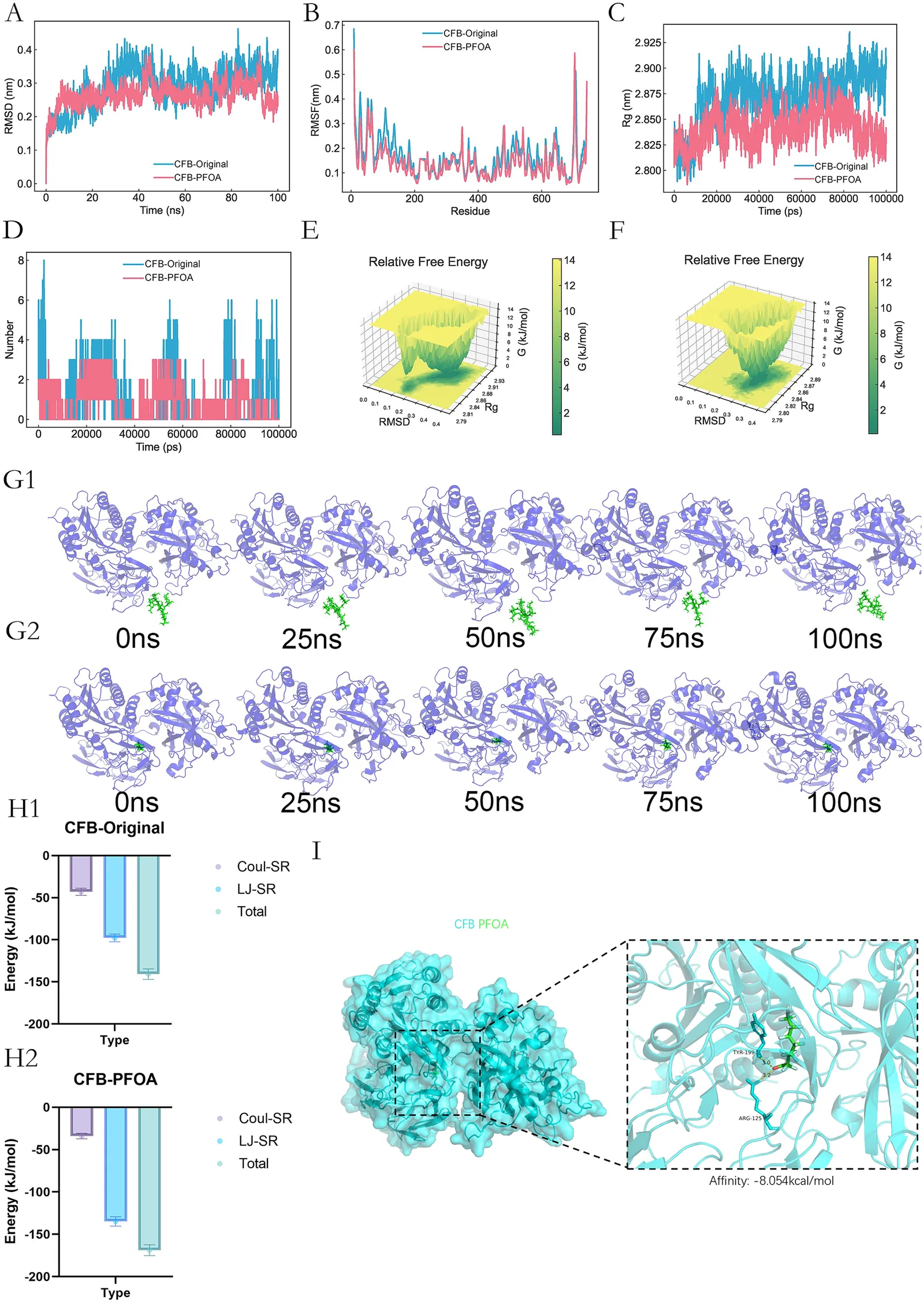
Molecular dynamics behavior of the predicted CFB–PFOA complex. **(A)** Backbone RMSD of the CFB–Original reference-ligand system and the CFB–PFOA system over the sampled 100-ns trajectories. **(B)** Per-residue RMSF profiles of the two simulated systems. **(C)** Time evolution of the radius of gyration (Rg) in the two simulated systems. **(D)** Hydrogen-bond contacts between CFB and PFOA during the sampled trajectory. **(E,F)** RMSD–Rg free-energy landscapes of the CFB–Original reference-ligand system **(E)** and the CFB–PFOA system **(F)**, illustrating the conformational space sampled during the simulations. **(G1,G2)** Representative snapshots at 0, 25, 50, 75, and 100 ns for the CFB–Original reference-ligand system **(G1)** and the CFB–PFOA system **(G2)**, showing retention of the predicted PFOA pose during the sampled trajectory. **(H1,H2)** Descriptive short-range Coulombic (Coul-SR), Lennard-Jones (LJ-SR), and total interaction-energy values for the CFB–Original and CFB–PFOA systems. The displayed variability reflects sampled trajectory frames and should not be interpreted as independent simulation replicates. **(I)** Selected docking pose of PFOA (green) within the CFB pocket, with a predicted docking score of −8.054 kcal·mol⁻1. The docking score and molecular dynamics data do not establish experimental binding or regulatory direction.

To explore the behavior of the predicted PFOA–CFB complex, we compared a CFB–Original reference-ligand system with the CFB–PFOA system over a single 100-ns trajectory. Within the sampled trajectory, the RMSD of the CFB–PFOA system reached an apparent plateau after approximately 15–20 ns and remained lower than that of the reference-ligand system during part of the simulation (Figure 3A). The radius of gyration was also modestly lower in the CFB–PFOA system during the sampled period (Figure 3C).

Residue-level RMSF profiles were broadly comparable between the two systems, with modest differences in several regions surrounding the selected pocket (Figure 3B). Recurrent hydrogen-bond contacts between PFOA and surrounding residues were observed during the trajectory (Figure 3D). The RMSD–Rg free-energy landscapes and representative structural snapshots further illustrated the conformational space sampled by the two computational systems (Figures 3E–G).

The predicted PFOA pose remained within the selected pocket during the sampled 100-ns trajectory, and the calculated short-range electrostatic and van der Waals interaction energies were negative (Figures 3H,I). However, because only a single 100-ns trajectory was analyzed and formal convergence validation was not performed, these observations do not establish complete equilibrium, long-term binding stability, direct physical binding under experimental conditions, or an activating or inhibitory effect on CFB enzymatic function. The computational results therefore provide a testable structural hypothesis rather than functional evidence of CFB regulation.

### 3.4 Interaction landscape and pathway enrichment of CFB

**Figure 4:**
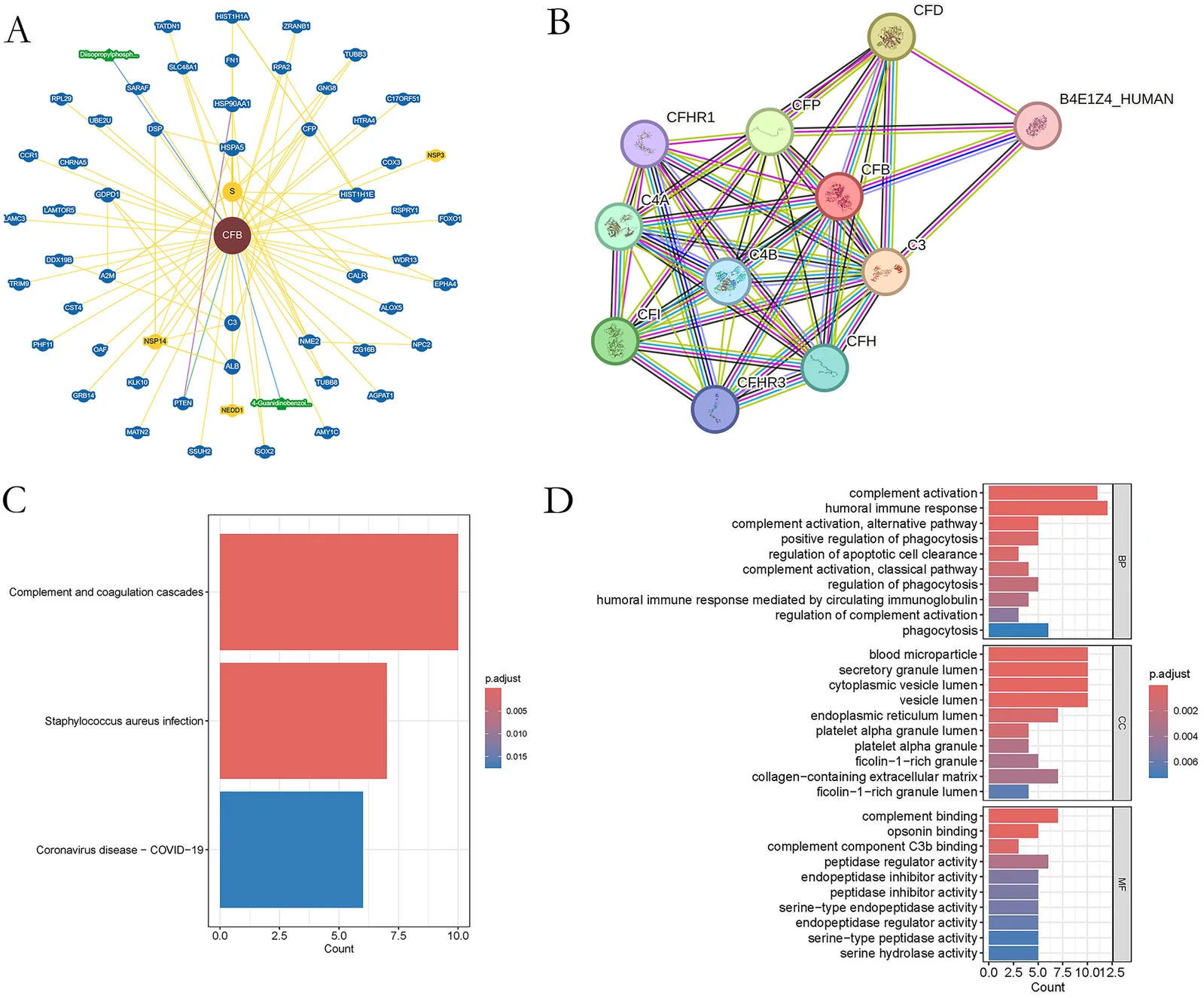
Interaction networks of CFB and pathway enrichment. **(A)** BioGRID-derived interaction map centered on CFB, showing experimentally reported binding partners. **(B)** STRING protein–protein interaction network of high-confidence CFB partners, highlighting core complement components. Edge colors reflect evidence channels. **(C)** KEGG pathway enrichment of the combined interactor set. Bar length denotes gene count, and color encodes adjusted *P* values. **(D)** GO enrichment summarized by ontology: biological process (BP), cellular component (CC), and molecular function (MF). Bar length denotes gene count, and color encodes adjusted *P* values.

To delineate the functional context of Complement Factor B (CFB), we compiled its interaction partners from two orthogonal resources. The BioGRID set captured experimentally reported interactors (Figure 4A), while STRING contributed high-confidence functional partners (Figure 4B). Integration of both sources highlighted a dense complement-centered subnetwork comprising core alternative-pathway enzymes and regulators—C3, C4A/C4B, CFD (factor D), CFP (properdin), CFH, CFI, and CFHR1/3—with CFB positioned as a central node. The STRING network displayed extensive interconnections among these proteins, consistent with a tightly coordinated amplification loop of the complement cascade.

Pathway over-representation analysis returned Complement and coagulation cascades as the top KEGG term (adjusted *P* < 0.05) (Figure 4C), followed by Staphylococcus aureus infection and Coronavirus disease–COVID-19. These results underscore CFB's role at the interface of innate immune defense, inflammation, and hemostasis.

GO enrichment further supported this interpretation (Figure 4D). In Biological Process (BP), terms related to humoral immune response, complement activation (including the alternative pathway), phagocytosis, and positive regulation of phagocytosis were significantly enriched. For Cellular Component (CC), interactors localized predominantly to secretory/platelet granule lumen, vesicle lumen, endoplasmic reticulum lumen, blood microparticle, and collagen-containing extracellular matrix, consistent with secretion and extracellular deployment of complement factors. In Molecular Function (MF), we observed enrichment of complement binding (including C3b binding), opsonin binding, peptidase regulator activity, and serine-type endopeptidase activity, reflecting the proteolytic and regulatory nature of complement amplification.

Collectively, the convergent interaction networks and enrichment signatures position CFB as a hub in complement-driven immunity with crosstalk to coagulation, providing a mechanistic framework for downstream studies (e.g., in atrial fibrillation) that interrogate inflammation-coagulation axes.

### 3.5 PFOA exposure, CFB expression, and AF-associated molecular markers in HL-1 cells

**Figure 5:**
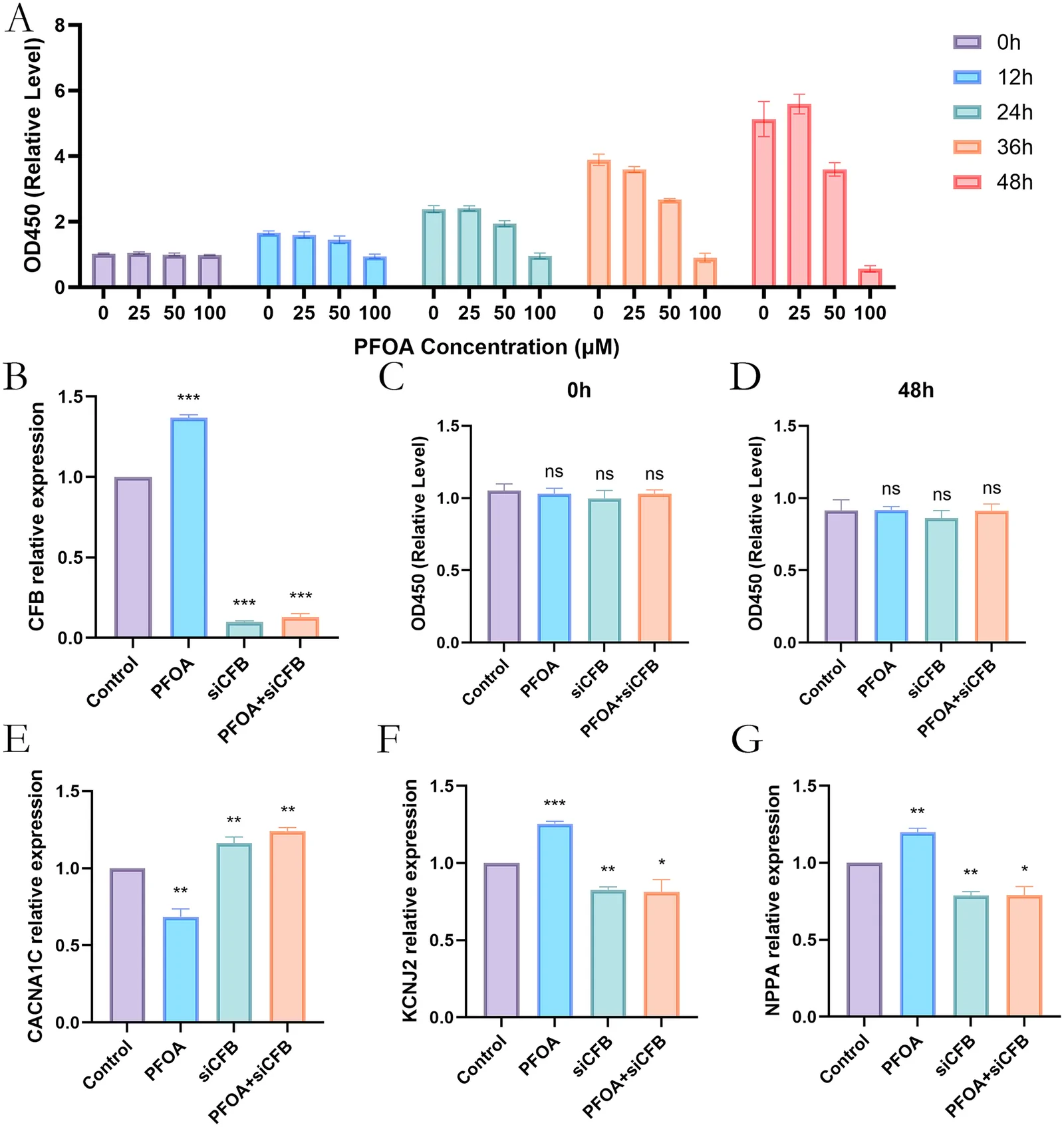
PFOA exposure, CFB expression, and selected AF-associated molecular markers in HL-1 cells. **(A)** CCK-8 screening across PFOA concentrations (0–100 µM) and times (0–48 h). A working condition of 25 µM PFOA for 48 h was selected because it did not significantly reduce CCK-8 viability under the tested conditions. **(B)** Relative Cfb mRNA expression in the Control, PFOA, siCFB, and PFOA + siCFB groups. **(C,D)** CCK-8 measurements in the four experimental groups at 0 h and 48 h. **(E–G)** Relative expression of the selected AF-associated transcripts Cacna1c (E), Kcnj2 **(F)**, and Nppa **(G)**. These markers do not by themselves establish an AF phenotype. Bars show mean ± SEM from ≥3 independent experiments. Significance: ns, not significant; * *p* < 0.05; ** *p* < 0.01; *** *p* < 0.001 (one-way ANOVA with Tukey's *post-hoc* test).

CCK-8 screening was used to select an *in vitro* working condition. Across the tested concentrations and durations, 25 μM PFOA for 48 h did not significantly reduce CCK-8 viability relative to the corresponding control and was therefore selected for the mechanistic experiments (Figure 5A). This condition should be interpreted as an experimental exposure and not as a surrogate for typical human serum concentrations.

qRT-PCR showed that PFOA increased Cfb mRNA expression in HL-1 cells, whereas Cfb-specific siRNA markedly reduced Cfb expression in both the absence and presence of PFOA (Figure 5B). No significant differences in CCK-8 viability were observed among the Control, PFOA, siCFB, and PFOA + siCFB groups at the analyzed time points (Figures 5C,D).

PFOA exposure decreased CACNA1C transcript levels and increased Kcnj2 and Nppa transcript levels (Figures 5E–G). CFB silencing attenuated the PFOA-associated changes in Kcnj2 and Nppa, whereas CACNA1C did not show a corresponding PFOA-dependent rescue pattern. These measurements demonstrate changes in selected AF-associated molecular markers but do not establish electrical remodeling or an AF phenotype.

Together, the data support an association between PFOA exposure, increased CFB expression, and alterations in selected AF-associated transcripts in HL-1 cells. They do not demonstrate functional complement activation or direct electrophysiological consequences.

### 3.6 Protein-level and immunofluorescence analyses in HL-1 atrial cardiomyocytes

**Figure 6:**
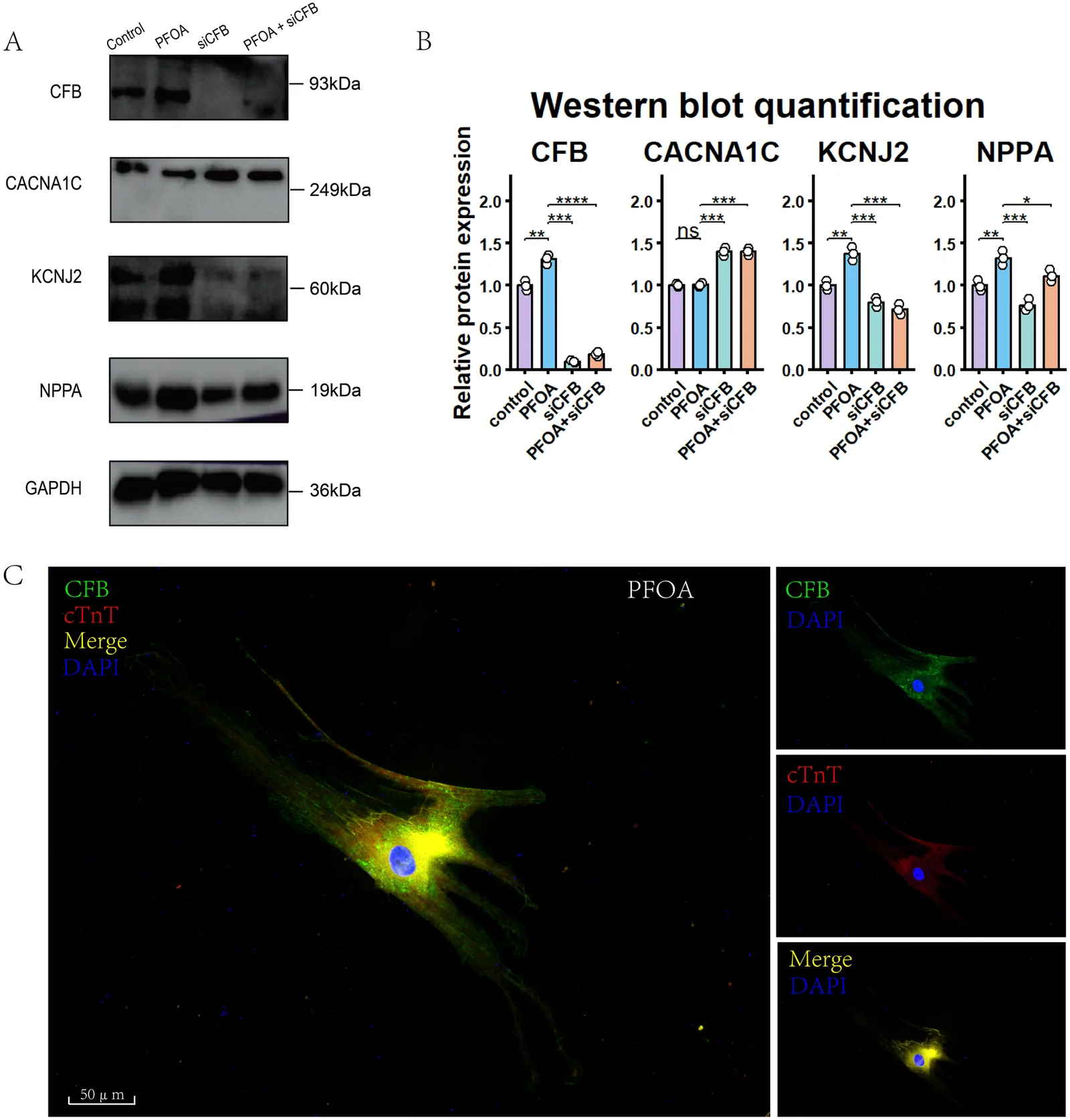
Western blotting and immunofluorescence analyses of CFB and selected AF-associated molecular markers in HL-1 atrial cardiomyocytes. **(A)** Representative Western blot bands of CFB, CACNA1C, KCNJ2, and NPPA in the Control, PFOA, siCFB, and PFOA + siCFB groups. GAPDH was used as the loading control. **(B)** Quantification of protein expression normalized to GAPDH. PFOA increased CFB, KCNJ2, and NPPA protein abundance, whereas CACNA1C was not significantly changed relative to Control. CFB silencing attenuated the PFOA-associated changes in KCNJ2 and NPPA. **(C)** Representative immunofluorescence images of PFOA-exposed HL-1 cells showing CFB immunoreactivity (green), the cardiomyocyte marker cardiac troponin T (cTnT; red), and DAPI-stained nuclei (blue). Yellow in the merged image indicates spatial overlap of the green CFB and red cTnT channels and is not a separate marker. Scale bar, 50 μm.

Western blotting was used to evaluate CFB and selected AF-associated molecular markers in the Control, PFOA, siCFB, and PFOA + siCFB groups (Figures 6A,B). PFOA increased CFB protein abundance, whereas CFB silencing markedly reduced CFB protein levels in both the absence and presence of PFOA.

PFOA increased KCNJ2 and NPPA protein abundance, while CACNA1C protein abundance was not significantly changed relative to Control. CFB silencing attenuated the PFOA-associated changes in KCNJ2 and NPPA. These protein-level changes are consistent with alterations in selected AF-associated molecular markers but are insufficient to establish a functional electrical-remodeling phenotype.

Immunofluorescence staining was performed to verify the cellular localization of the detected CFB signal. In PFOA-exposed HL-1 cells, cytoplasmic CFB immunoreactivity (green) was detected within cTnT-positive cardiomyocytes (red), and overlap of the green and red channels appeared yellow in the merged image (Figure 6C). DAPI-stained nuclei were blue. This result supports the presence of CFB immunoreactivity in the HL-1 cardiomyocyte model under the tested exposure condition.

## 4 Discussion

Our study used an integrated, hypothesis-generating framework to prioritize CFB as a candidate molecule potentially associated with PFOA exposure and AF-related molecular changes. The network toxicology, exploratory MR, molecular simulation, and HL-1 cell results provide complementary but distinct levels of evidence. These findings support further investigation of CFB-associated responses to PFOA but do not establish direct causality, functional complement activation, or a functional AF phenotype.

The network toxicology analysis was used for exploratory candidate prioritization. Because CTD, STITCH, and SwissTargetPrediction contain different types of curated associations and computational predictions, the integrated candidate set should not be interpreted as a collection of experimentally confirmed direct PFOA-binding targets. Moreover, the network intersection itself does not determine regulatory direction or establish causality.

The elevation of CFB in the AF group aligns with growing recognition that inflammation and complement activation play crucial roles in atrial remodeling and arrhythmogenesis (42, 43). While much attention has been paid to classical inflammatory cytokines (e.g., IL-6, TNF-α), the complement system represents a parallel inflammatory cascade that amplifies immune responses and contributes to tissue injury (44, 45). CFB, as the essential component of the alternative complement pathway, mediates amplification of C3 cleavage and propagation of downstream effector functions including chemotaxis (via C5a), vascular permeability, and membrane attack complex formation (46, 47). In cardiac tissue, excessive complement activation has been associated with myocardial infarction, myocarditis, and ischemia–reperfusion injury (48, 49). The enrichment results place CFB within established complement-related biological pathways; however, the present study did not directly measure complement activation or demonstrate functional atrial electrical remodeling.

Mechanistically, CFB expression and activity have been linked to various cardiovascular pathological states. Previous studies have reported that complement activation contributes to atrial fibrosis by recruiting pro-inflammatory cells and stimulating fibroblast differentiation (50). In cardiac surgery models, increased CFB levels postoperatively correlate with higher incidence of postoperative AF (51). Experimental evidence from CFB-deficient mice suggests that blockade of this pathway confers protection against ischemic injury and adverse cardiac remodeling. Furthermore, in metabolic disease states, such as type 2 diabetes, elevated CFB levels in adipose tissue have been associated with macrophage M1 polarization, chronic inflammation, and vascular dysfunction (52). Collectively, these data suggest that CFB is not merely a marker of systemic inflammation but a functional driver of immune–cardiac interaction and structural remodeling—a key substrate for AF maintenance.

Our molecular docking and dynamics simulations provide a computational structural hypothesis for a potential PFOA–CFB interaction. Docking results revealed favorable binding of PFOA within a positively charged groove near the catalytic region of CFB, stabilized by hydrogen bonding and electrostatic interactions between the carboxylate group of PFOA and basic residues (e.g., Arg) on the protein surface. The hydrophobic perfluorinated chain of PFOA also nestled into nonpolar pockets, reinforcing complex stability. Subsequent 100 ns molecular dynamics simulations confirmed the persistence of the PFOA–CFB complex, with root-mean-square deviation (RMSD) values stabilizing after initial equilibration, indicating a structurally stable interaction. These findings suggest that PFOA may alter the conformational landscape or enzymatic efficiency of CFB, either enhancing or dysregulating its role in complement activation. While PFOA is not a classical ligand, its chemical properties—namely, lipophilicity, polarity, and molecular rigidity—render it capable of nonspecific yet functionally relevant interactions with diverse protein targets.

From a translational perspective, CFB and its downstream complement components may serve as candidate biomarkers for environmental exposure and cardiac risk. Monitoring CFB levels in serum or atrial tissue—possibly alongside CRP or C3a—could aid in assessing AF susceptibility in populations with elevated PFAS burden. Moreover, targeting the CFB–complement axis may offer a novel therapeutic avenue. Selective complement inhibitors, such as factor B antagonists or C3 convertase blockers, have shown promise in renal and autoimmune diseases and may be repurposed for cardiac inflammation contexts (53). Importantly, such an approach may be particularly effective in individuals with toxin-induced inflammation where upstream immune triggers are less amenable to traditional therapies (e.g., rate or rhythm control medications).

Although public single-cell RNA-sequencing data from the normal human heart indicated that basal CFB expression was preferentially enriched in fibroblast and stromal populations, our cellular experiments demonstrated that CFB was detectable in HL-1 atrial cardiomyocytes at both the mRNA and protein levels. Immunofluorescence staining further identified CFB immunoreactivity within cTnT-positive, PFOA-exposed HL-1 cells. These findings support the capacity of atrial cardiomyocytes to express CFB under toxicological stress. Nevertheless, the present results do not indicate that cardiomyocytes are the predominant source of CFB in the intact heart. Fibroblast-derived and circulating CFB may also contribute substantially to complement-associated cardiac responses *in vivo*.

Several limitations should be acknowledged. First, the HPA single-cell data indicate that basal CFB expression in the normal human heart is preferentially enriched in fibroblast and stromal populations. Although CFB was detectable in PFOA-exposed, cTnT-positive HL-1 cells, the present monoculture model does not reproduce fibroblast–cardiomyocyte paracrine interactions or establish cardiomyocytes as the predominant source of cardiac CFB *in vivo*. Second, increased CFB mRNA and protein expression does not demonstrate functional activation of the alternative complement pathway because CFB cleavage, C3 convertase activity, C3a/C5a generation, and C5b-9 formation were not measured. Third, changes in CACNA1C, Kcnj2, and Nppa expression represent alterations in selected AF-associated molecular markers and do not establish functional electrical remodeling or an AF phenotype. Electrophysiological studies, primary or human atrial cardiomyocytes, and *in vivo* models would be required for functional validation. Fourth, 25 μM PFOA is substantially higher than typical circulating concentrations in the general population. Although this concentration did not significantly reduce CCK-8 viability under the present conditions, nonspecific, sublethal, or off-target effects cannot be excluded, and the findings should not be interpreted as quantitatively representative of real-world exposure. Fifth, the network toxicology analysis was exploratory and combined heterogeneous sources of curated associations and computational predictions. The intersection does not independently establish direct binding, regulatory direction, or causality. Sixth, the MR analysis lacked a complete set of instrument-strength, horizontal pleiotropy, and leave-one-out sensitivity assessments and should therefore be interpreted as exploratory genetic support rather than proof of causality. Finally, the molecular dynamics analysis was limited to a single 100-ns trajectory without replicate simulations or formal binding-free-energy convergence validation. The computational results therefore support only a testable structural hypothesis. In addition, chronic low-dose exposure conditions were not evaluated in the present study.

In summary, this study prioritizes CFB as a candidate molecule potentially associated with PFOA exposure and AF-related molecular changes. PFOA increased CFB expression and altered selected AF-associated markers in HL-1 cells, while CFB silencing attenuated these changes. These exploratory findings generate a testable hypothesis but do not establish functional complement activation, direct causality, or a functional AF phenotype.

## Data Availability

The datasets presented in this study can be found in online repositories. The names of the repository/repositories and accession number(s) can be found in the article/Supplementary Material.
